# Augmenting locomotor perception by remapping tactile foot sensation to the back

**DOI:** 10.1186/s12984-024-01344-7

**Published:** 2024-04-27

**Authors:** Atena Fadaei Jouybari, Nathanael Ferraroli, Mohammad Bouri, Selim Habiby Alaoui, Oliver Alan Kannape, Olaf Blanke

**Affiliations:** 1 Laboratory of Cognitive Neuroscience, Faculty of Life Sciences, Neuro-X Institute, Swiss Federal Institute of Technology (EPFL), Geneva, 1012 Switzerland; 2 REHAssist Group, EPFL, Station 9, STI IMT MED, Lausanne, Switzerland; 3 Virtual Medicine Center, HUG-NeuroCentre, Department of Clinical Neurosciences, University Hospitals Geneva, Geneva, Switzerland

**Keywords:** Sense of agency, Locomotion, Tactile feedback, Motor awareness, Remapped touch, Human-machine-interaction

## Abstract

**Background:**

Sensory reafferents are crucial to correct our posture and movements, both reflexively and in a cognitively driven manner. They are also integral to developing and maintaining a sense of agency for our actions. In cases of compromised reafferents, such as for persons with amputated or congenitally missing limbs, or diseases of the peripheral and central nervous systems, augmented sensory feedback therefore has the potential for a strong, neurorehabilitative impact. We here developed an untethered vibrotactile garment that provides walking-related sensory feedback remapped non-invasively to the wearer’s back. Using the so-called FeetBack system, we investigated if healthy individuals perceive synchronous remapped feedback as corresponding to their own movement (motor awareness) and how temporal delays in tactile locomotor feedback affect both motor awareness and walking characteristics (adaptation).

**Methods:**

We designed the system to remap somatosensory information from the foot-soles of healthy participants (*N* = 29), using vibrotactile apparent movement, to two linear arrays of vibrators mounted ipsilaterally on the back. This mimics the translation of the centre-of-mass over each foot during stance-phase. The intervention included trials with real-time or delayed feedback, resulting in a total of 120 trials and approximately 750 step-cycles, i.e. 1500 steps, per participant. Based on previous work, experimental delays ranged from 0ms to 1500ms to include up to a full step-cycle (baseline stride-time: µ = 1144 ± 9ms, range 986-1379ms). After each trial participants were asked to report their motor awareness.

**Results:**

Participants reported high correspondence between their movement and the remapped feedback for real-time trials (85 ± 3%, µ ± σ), and lowest correspondence for trials with left-right reversed feedback (22 ± 6% at 600ms delay). Participants further reported high correspondence of trials delayed by a full gait-cycle (78 ± 4% at 1200ms delay), such that the modulation of motor awareness is best expressed as a sinusoidal relationship reflecting the phase-shifts between actual and remapped tactile feedback (cos model: 38% reduction of residual sum of squares (RSS) compared to linear fit, *p* < 0.001). The temporal delay systematically but only moderately modulated participant stride-time in a sinusoidal fashion (3% reduction of RSS compared a linear fit, *p* < 0.01).

**Conclusions:**

We here demonstrate that lateralized, remapped haptic feedback modulates motor awareness in a systematic, gait-cycle dependent manner. Based on this approach, the FeetBack system was used to provide augmented sensory information pertinent to the user’s on-going movement such that they reported high motor awareness for (re)synchronized feedback of their movements. While motor adaptation was limited in the current cohort of healthy participants, the next step will be to evaluate if individuals with a compromised peripheral nervous system, as well as those with conditions of the central nervous system such as Parkinson’s Disease, may benefit from the FeetBack system, both for maintaining a sense of agency over their movements as well as for systematic gait-adaptation in response to the remapped, self-paced, rhythmic feedback.

## Background

In parallel to assistive technology (AT) advancing beyond passive, mechanical engineering solutions to technological devices with embedded computing capacities and (bi-directional) neural interfaces [1, 2], so too has there been a shift in scientific thinking concerning once exclusively biological or psychological concepts of embodiment and body representation. Concepts such as ownership, referring to the sensation that our body belongs to ourself [3], and the sense of agency, that is, the feeling of being the author of and in control of your actions [4], are becoming common parlance within the AT community and used as metrics for the evaluation of these devices. Like earlier investigations in the cognitive sciences, these studies often rely on brief, easily quantifiable, and goal-directed actions, often with mediated outcomes. However, as we argue below, key insights may be gained by considering continuous movements (not just of the upper-limbs) and automatic motor compensation (outside of a goal-directed action). Furthermore, as in the case of patients with amputations or sensory neuropathies where natural sensory feedback is compromised, understanding how feedback may be augmented such that it is automatically integrated into sensorimotor control and how this contributes to ownership and agency is an area of both conceptual and translational interest [5, 6]. An important differentiator for movement perception, is based on perceiving and recognising this feedback as originating from one’s own as opposed to someone else’s body or a stereotyped movement, and this information may potentially feed into motor adaptation [7–9]. Accordingly, one motivating factor for this study was to determine to what extent sensorimotor feedback is perceived as self-generated and how this affects motor behaviour (adaptation). Based on our prior work on haptic vests [10, 11], we here developed a vibrotactile system to systematically evaluate participants’ perception of locomotor-feedback in relation to potential adaptation of their gait. To this end, the FeetBack system enabled us to non-invasively remap step-related feedback from participants’ foot soles to their own back during natural over-ground walking. The tactile sensation of the stance phase, from heel-strike to toe-off, is thereby remapped to the participant’s back using vibrotactile apparent movement (VAM). By modulating the timing-onset of the locomotor feedback we could quantify both participants’ awareness of their movements as well as the effects of the tactile feedback on sensorimotor control and adaptation.

### Tactile feedback for locomotion

Wearable biofeedback systems have been used to provide artificial tactile feedback in the form of simple vibrations during walking. Early studies with such devices have demonstrated that rhythmic tactile stimulation during locomotion may improve gait characteristics in Parkinson’s Disease [12], stroke [13], and hemi- or paraplegic patients [14], as well as lower-leg amputees [15]. Such approaches are interesting for three main reasons; for one they may be more practically integrated without interfering with auditory or visual function; for another, they can be “internally” paced, based on the participants on-going movements (as opposed to external rhythmic cueing); and finally, tactile stimulation can be used to augment somatosensory and proprioceptive feedback that can be impaired in patient populations and therefore not correctly integrated into on-going motor control [16]. As discussed in the following paragraphs, previous research in cognitive neuroscience has demonstrated how spatial and temporal mismatches introduced into the feedback not only inform us about patients’ motor awareness [17] but also lead to systematic sensorimotor adaptation that could potentially be exploited for rehabilitation purposes [18].

Motor Awareness and the Sense of Agency.

Although research in human neuroscience has predominantly focused on strictly pre-defined actions of the upper-limbs, most of the movements we perform over the course of a day are neither immediately goal-directed nor do they result in a consciously “desired outcome” [19]. For instance, we may adjust our posture after being stationary for an extended period of time, we may shift our weight to maintain our balance, or we may just be walking without an immediate target or particular goal, in the sense of a physical location. At the same time, we are in control of these actions and perceive them as our own; we perceive a sense of agency (SoA) for these actions [8].

Nonetheless, mirroring research on human sensorimotor control, SoA research has predominantly focused on brief upper-limb movements directed at specific target locations [20–26]. By introducing angular biases in visual feedback such studies have outlined how accurately participants can monitor their movements. We refer to this insight into our on-going movement as Motor Awareness (MA). Studies such as these have reliably reported that MA is limited. Movement feedback with angular deviations of 6.5°-15° is thus judged to not be deviated, even as participants subconsciously perform motor corrections [20, 23, 27, 28]. This limit of MA, constitutes an important aspect of our sense of agency in relation to sensorimotor control. It complements other aspects such as ownership over an action (“I am performing this action”), and action intentionality (“This is the action I planned.”).

Next to spatial deviations, studies that provided temporally manipulated visual feedback (hand movements) demonstrated that MA is further limited to mismatches delayed by more than150-200ms [20, 23, 25]. This line of research is often extended to mediated action outcomes such as the occurrence of a tone after a button-press [29–32], an approach further applied to investigate voluntary action and intention [33–35]. These studies thus investigated SoA by focusing on the outcome of mediated actions rather than conscious monitoring of the underlying movement itself (MA). Any judgment at the end of such a trial may therefore be skewed by the outcome or in some cases even be revised based on altered feedback [36].

### Motor awareness for locomotion

A complementary approach to studying SoA via MA has consequently been to focus on continuous and partially automated movements such as drawing [28, 37, 38], locomotion [39–44], or even respiration [45–49]. Rather than relying on the outcome of the motor task these studies focus on the level of conscious access that participants have for their movements. The spatiotemporal thresholds reported in these studies, that is, the psychometrically determined point of subjective equality where 50% of the deviated or delayed trials are judged to be veridical, are comparable to those of goal-directed tasks (within the range of 150-200ms) [42, 44]. In the case of locomotion, which is cyclic, not usually immediately goal-directed, and generally considered a highly automatic and unconscious action [50, 51], participants not only showed high MA for real-time trials but also in trials were the feedback was delayed by a full step-cycle and therefore “re-synchronised”. For auditory feedback, this high MA was also reported in the case of delays of half a step-cycle, even though this feedback was left-right reversed. Participants would hear a lateralized left heel-strike at the time of the actual right heel-strike and vice versa, indicating the importance of temporal information. This applies to both gait-awareness and gait-regulation: participants in such studies unconsciously and automatically adapted their movements depending on the spatial or temporal mismatch in the feedback [42, 44]. As discussed later this may also be linked to syncopation between the rhythm of the actual versus the feedback walking patterns [52]. Unlike for the aforementioned goal-directed movements, where such a compensation is required to complete the task, adaptation in the continuous task is neither required nor does it affect the outcome of the task, demonstrating that gait-related feedback may automatically be integrated in feed-forward motor control and potentially targeted for neurorehabilitation and re-education.

**Fig. 1:**
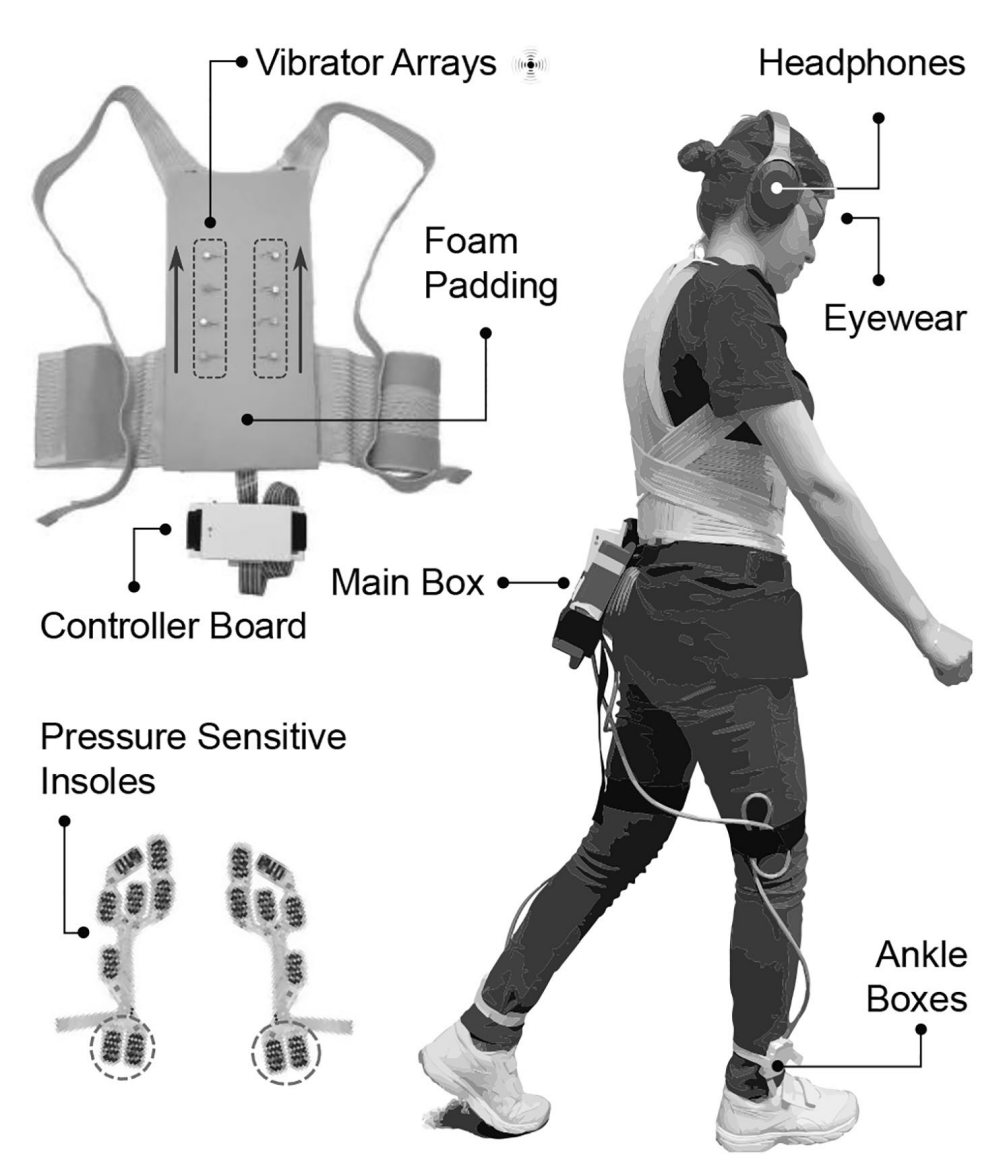
FeetBack system on the user and its components. FeetBack system remaps the foot rolling sensation under the user’s feet (lateralized feedback on the stance) onto the skin of the back. It includes two main parts: a wearable gait measurement system and a torso-worn vibrotactile display. The wearable gait measurement system detects the user’s step. It consists of pressure-sensitive insoles under the user’s feet, two ankle boxes, and the main control board attached to the participants’ lower back. The two force-sensitive sensors that are located at the heel (specified with dashed circles) were used to detect heel-strike. The vibrotactile display provides moving sensations (i.e., VAM) on the participant’s back and includes two vertical vibrator arrays attached to foam, a torso-worn vest, and the controller board. Feedback on the stance was provided from down to up (solid black arrows)

### The FeetBack system

While prior studies using auditory and visual stimuli have thus delineated important aspects of MA for locomotion, arguably the most fundamental consequence of our movements are proprioceptive [53] and tactile in nature [54], such as ground reaction forces encountered at each heel-strike. To quantify the integration of tactile feedback to participants’ SoA we here investigated MA for remapped tactile feedback. To this aim, we developed the FeetBack system, a vibrotactile system that provides non-invasive, step-related, remapped feedback during free-walking onto the back of the wearer, Fig. 1. We adapted previous paradigms for conscious gait monitoring via auditory [42] and visual [44] cues to touch feedback during walking. This wearable, untethered tactile remapping system allowed us to introduce a specific range of temporal delays between participants’ actual, on-going foot-steps and step-related tactile cues provided onto the skin of their back [10, 11]. We hypothesised to observe systematic modulation in MA depending on the delay, with strongest MA ratings for both real-time feedback, as well as feedback with delays closest to the duration of the participants gait-cycle (re-synchronised feedback). We were further interested to see if this would evoke a systematic modulation of participants’ walking speed as a function of temporal delay as described by [42] and [44], demonstrating the effect of cognetic interfaces on the robot-controlled bodily action-perception cycle [55].

## Methods

### Participants

A total of 29 healthy participants (18 female) with normal or corrected-to-normal hearing and vision, ranging in age from 21 to 36years (M = 26.86y, SD = 4.6y), were recruited in the experiment. They were all right-handed with no history of known orthopaedic, metabolic, or neurological impairment or painful condition that might alter walking. They did not report having scar-tissue on the back that could have influenced sensitivity to the tactile stimuli. All participants were naïve to the purpose of the study and gave written informed consent before participating in the experiment. The study protocol was approved by the local ethical committee.

### FeetBack system

We remapped somatosensory information from the heel-strikes of participants, walking over-ground at their preferred speed, onto the surface of their back using the FeetBack system (see Fig. 1). To this end, two linear arrays of vibrators were mounted on the sides of the back, each remapping heel-strike and footfall of the ipsilateral leg. We used vibrotactile apparent movement to induce a movement sensation on the back [56], similar to the heel-strike pattern. VAM can be invoked through activating two or more vibrators, sequentially with specific timing parameters, namely duration of stimuli (DOS, per vibrator) and stimuli onset asynchrony (OA, between two vibrators). As a result, the discrete stimulation is perceived as if moving continuously from one position to another [57, 58]. The intrinsic delay of the system, from detecting the heel-strike to providing tactile feedback, was 60ms.

A pilot study with *N* = 5 participants was conducted to I) determine appropriate OA and DOS parameters that would induce VAM with the FeetBack system and ii) select an appropriate VAM profile. VAM profiles were evaluated in a 2 × 2 design comparing the phase of the gait-cycle (VAM during stance-phase vs. swing-phase ) and the perceived VAM direction (upward vs. downward). Participants reported that the upward VAM presented a better remapped experience of the natural heel-strike and stance phase. VAM duration, i.e., the time of presenting the VAM with each linear array of vibrators, was fixed at 405ms, such that stimulation stopped prior to the toe-off and swing phase. This results from summing the DOS across the four vibrators and subtracting the three overlaps (the difference between DOS and OA). This value was fixed as the VAM could not be calculated for each step in real-time and would further introduce a step-to-step variability that could interact with the main independent variable, the temporal delay. Based on this study, VAM timing parameters were set to DOS = 150ms and OA = 85ms.

### Wearable gait measurement system

Pressure-sensitive insoles were used to measure the stride time, defined as the time elapsed between two consecutive heel-strikes of the same foot (HD-FSR 002 by IEE S.A., Contern, Luxembourg.; Fig. 1). Each insole contained eight force-sensitive resistors (locations: 2 at the heel, 1 at lateral mid-foot, 3 at the ball of the foot, 2 in the front) capable of recording a pressure range from 100mbar to 6 bar. Pressure insoles were provided in two sizes (Medium and large). Changes in pressure, represented by changes in resistance were read out by two boxes mounted to the ankles and containing the electronics (one Wheatstone Bridge per sensor, and an AD converter). Pressure data is sent to the main box, mounted on the back, using SPI (Serial Peripheral Interface) through a shielded cable.

The mainboard served as the central computing unit, collected all sensor data and communicated with the host PC through Wi-Fi (receive/send; sampling time of 10 msec). It included a BeagleBone Black (BBB, a single-board computer, Beagle-Board.org Foundation), an Inertial Measurement Unit to record acceleration and gyroscope information (not used in the current study), a WiFi module (TP-LINK WLAN-N-USB adapter) and a battery (power bank, 3000 mAh) making the system fully portable.

Torso-worn vibrotactile display.

To provide VAM on the participants’ back, two vertical arrays of coin-shaped, eccentric rotating mass (ERM) vibrators (310-003, Precision MicroDrive; body diameter: 10 mm; body length: 3.4 mm; weight: 1.1 gr) were attached to a 20 mm-thick foam (Softpur polyurethane foam) with a horizontal distance of 110 mm using snap fasteners (see Fig. 1). There were four vibrators in each array (inter-tactor distance of 40 mm). Fasteners were respectively glued to vibrators and foam. The vibrator foam was fixed to a fully elastic, posture-corrector brace using Velcro straps. The posture-corrector firmly keeps vibrators against the skin while allowing the user to move conveniently. The ERMs are controlled by a 5 V haptic motor driver (DRV2605, Texas Instruments), resulting in a vibration frequency of 175 Hz, and an acceleration of 1.3G. Haptic drivers were controlled with an STM32F407 microcontroller, connected to the host PC using a Bluetooth module (HC-05). The controller board (Fig. 1) can be fully portable (battery-powered) or tethered for more extended studies.

A customized GUI was implemented in the Qt platform, a free and open-source platform, to provide a convenient interface for controlling the experiment. The GUI received pressure data from the gait measurement computing units via the WiFi connection and provided live plotting of data together with other functionalities allowing the experimenter to monitor the ongoing study in real-time. It further allowed adjusting vibrator parameters (e.g., intensity, DOS, OA) and sending commands to the vibrotactile display for presenting VAM stimuli.

### Paradigm

**Fig. 2:**
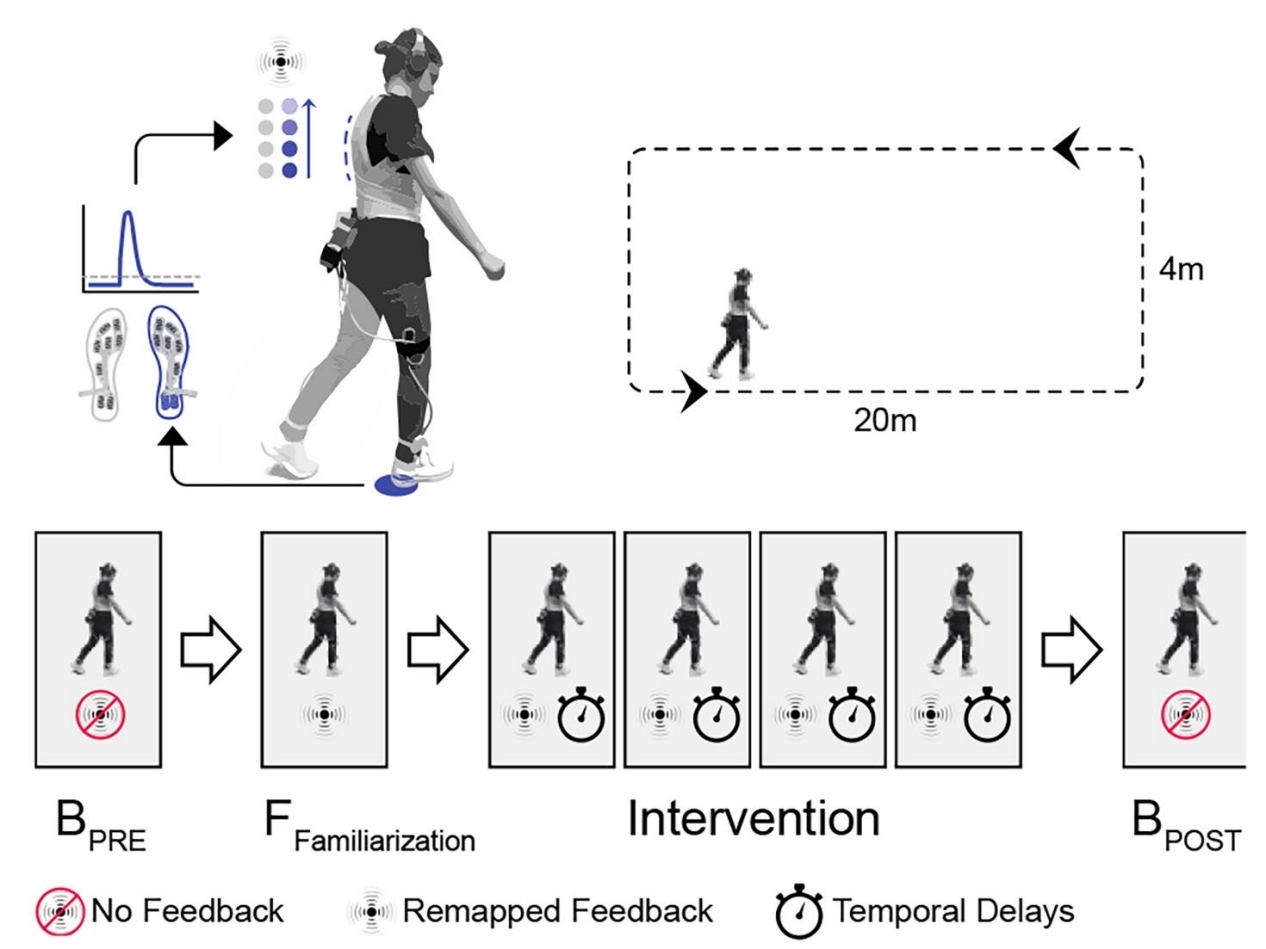
FeetBack Stimulation and Experimental Protocol. (top left to right) The system logs a heel-strike when the force-sensitive sensor crosses an individualized threshold. It subsequently triggers the vibrotactile apparent movement stimulation on the corresponding side of the back, either in real-time or with an experimental delay. Participants walked along a 20 m by 4 m rectangle, so that they could complete one trial while walking along one of the longer sides. (lower left to right) To begin with, participants were asked to walk freely, at their preferred speed, so that baseline walking characteristics could be determined (B_PRE_). Subsequently, participants wore the FeetBack system, personalized to the baseline gait characteristics, and received real-time tactile feedback about their on-going movements, triggered by each heel-strike (F := Familiarization). The main experiment was broken into four blocks (Intervention). Here, participants had to complete individual trials in which feedback was presented either in real-time (60 ms system delay) or randomly delayed by up to 1,500 ms. After each trial, participants reported their Motor Awareness by replying to the Yes/No forced-choice question: “Did the feedback you felt on your back exactly correspond to the walking you just performed?”

The experiment was conducted using a within-participants repeated measures design. As illustrated in Fig. 2, there were two baseline blocks: prior to (pre-baseline: no tactile feedback, B_Pre_), and after the intervention (post-baseline: no tactile feedback, B_Post_). Familiarization and pre-baseline blocks allowed us to establish points of reference to calculate the stride time alterations that were used in the intervention. The intervention consisted of trials with non-delayed and delayed feedback (i.e., delay as a with-in participant variable), including eleven levels of delay (ranging from 0ms to 1500 ms at increments of 150 ms). We also included catch trials that consisted of a noisy tactile sensation to assess the extent to which participants responded to perceived tactile feedback on their back rather than using other response strategies. Each condition was repeated ten times resulting in a total of 120 trials per participant. All delay conditions were presented randomly during the intervention, in a total of four blocks. MA was assessed at the end of each trial, and participants were asked to respond (“yes,” “no”). Based on previous work [42, 44], we asked the participants: “Did the feedback you felt on your back exactly correspond to the walking you just performed?”. The ratio of “yes” responses, given via button press at the end of each trial, was analyzed and reported. To capture any alterations in participants’ walking pattern, influenced by the different feedback conditions, participants’ stride time values were recorded. We carried out a post-baseline condition to assess whether there was any influence of the intervention on subjects’ gait (e.g., habituation, fatigue, etc.) that persisted beyond the experimental conditions.

### Procedure

The experimental procedure is illustrated in Fig. 2. After providing informed consent, participants put on a fitted T-shirt, the actuated vest, and the wearable gait measurement system. Three different sizes (37, 40, 43 EU size) of the same brand shoes, including the corresponding size shoe soles (M/L), were provided. Participants also wore occluding eyewear (SKLZ Court Vision Basketball Dribbling Goggles), preventing them from seeing their legs while walking. In addition, participants received white noise through noise-canceling headphones (WH-1000XM3, Sony) to attenuate potentially distracting ambient sounds and to mask any acoustic cues that might be related to activation of vibrators or their foot-steps. A single or multiple beeps indicated the start and end of each block respectively, both for baseline and trial blocks.

After donning the experimental equipment, participants were asked to walk on a predefined rectangular walking path of 4 × 20 m (marked on the floor with red tape) in a large open space for as many iterations as they wanted, but at least one full turn. This allowed participants to habituate to the experimental setup and walking path. We also checked whether pressure data from the foot soles were correctly acquired and recorded by the software at this time. Participants were instructed to walk (counterclockwise) at their preferred speed throughout the whole experiment (as if they were “taking a stroll along a foot-path”). They were asked to maintain their walking speed through turns as much as possible.

In the first of two baseline blocks (B_Pre_; see Fig. 2), participants were asked to complete one full turn around the rectangular path. Next they completed a Familiarization block (see Fig. 2), where they received real-time tactile feedback while walking for one loop. This was followed by a training block and the main experimental block. Finally, a second baseline block was completed (B_Post_).

Trials in the training block and in the main experimental block lasted 7 s, resulting in an average of 6.3 step-cycles (± 0.7 steps; SD) per trial. To minimize any potential effect of turning on the stride time, participants were instructed to start each trial when they started walking down the length of the space by pressing the start button, such that they could finish each trial in a single straight without turning. Training lasted approximately 5 min, including three different delays of zero, 300 ms, and 600 ms, each repeated three times. In this way, participants familiarized themselves with the task and subsequent question, which was answered via two response buttons. The main experimental block was split into four sub-blocks between which participants had the opportunity to be seated and take a drink. At the end of the experiment, participants were asked to comment freely on their experience and the experiment (debriefing; Fig. 2).

### Analysis

#### Motor awareness

We computed MA for each delay as the ratio of “yes”-responses over all trials. Participants that responded “yes” to three catch trials or gave “yes” responses for more than 90% of highly out-of-phase trials (e.g., 1/2 cycle delay) were excluded (3 participants). A total of 26 participants (17 female, aged between 22 and 36 years, mean = 27 years, SD = 4.5 years) were thus inluded in the analysis.

A sub-analysis determined the psychometric thresholds, that is the point of subjective equality, indicating the delay at which a participant would respond “yes” in 50% of cases, based on trials from 0ms to 600ms delays. Thresholds for six participants could not be extracted. Two had MA ratings of just 50% (yes responses) for non-delayed feedback, and four never rejected more than 50% (no responses) for delays up to 600ms such that a psychometric function could not be fit to the data. Hence, temporal MA threshold are reported in the results section for 20 participants.

#### Gait parameters

Each gait cycle started with the heel strike as detected by the two force-sensitive resistors located at the heel (Fig. 1, specified with dashed circles) using a personalized threshold. The threshold was set manually based on a preliminary recording of the participants gait. Stride-times were calculated as the time interval between two successive heel strikes of the same foot. Stride-time was separately calculated for each leg and only complete cycles (for each leg) were included in the average for each trial. Cycles shorter than 900ms or longer than 1500ms were excluded [59]. Stride-time calculation was processed online via the GUI and recorded for statistical analysis. The average stride-time for the left and right leg was employed as each trial’s stride time for the final analysis. We excluded trials with a stride time that deviated > 3 SD from the median (Median Absolute Deviation criteria with the factor of 3 [60]). On average, only 0.73 of 110 trials (per participant) were rejected.

Moreover, to compare stride time alterations in baseline and intervention blocks, mean stride time (M_ST_) and the stride time coefficient of variation (CV_ST_; i.e., the ratio of the standard deviation over the absolute mean) were calculated for each individual participant, across blocks. In order to diminish between-participants variability, stride time deviations (i.e., the difference between the average stride time of an individual trial and the average stride time during the intervention for each participant) were used to assess stride time modulation in the intervention session.

### Statistical analysis

Analyses were performed in either JASP [61] and R [62] running in the RStudio environment. The normality of the residuals together with linearity and homogeneity of variance was checked. Repeated-measures ANOVA were conducted for MA and stride time deviation data, with Delay as independent variable (11 levels). Posthoc comparisons were conducted using Tukey’s honest significant difference test (Tukey HSD). Significance was set at *p* < 0.05.

An rmANOVA was performed to assess differences in M_ST_ and CV_ST_ between Blocks (B_Pre_, familiarization, intervention, B_Post_). We further examined the effect of presenting tactile feedback on gait parameters by collapsing data into feedback (including familiarization and intervention blocks) versus no-feedback (including B_Pre_, and B_Post_ blocks) groups. A simple paired t-test was used to assess the overall effect of tactile feedback compared to no tactile feedback.

Nonlinear regressions function were used to fit harmonic function to both MA and stride time deviation data, in R (nls(); nonlinear least squares) and determine the coefficients of the parameters in the model (MA rating: cosine function; stride time deviation: sine function). The linear and nonlinear models for MA and stride time deviation data were compared in R with ANOVA, using Aikake and Bayes Information Criteria (AIC and BIC, respectively) [63, 64].

Temporal thresholds were determined by fitting a cumulative Gaussian to the MA responses for trials with 0-ms to 600-ms delays with the published psignifit toolbox [65, 66] for MATLAB (MathWorks, Natick, MA). This toolbox enforces bootstrapping algorithms and weighs the individual data points based on the number of valid trials per stimulus intensity. All thresholds reported here reflect the 50% point of subjective equality.

**Fig. 3:**
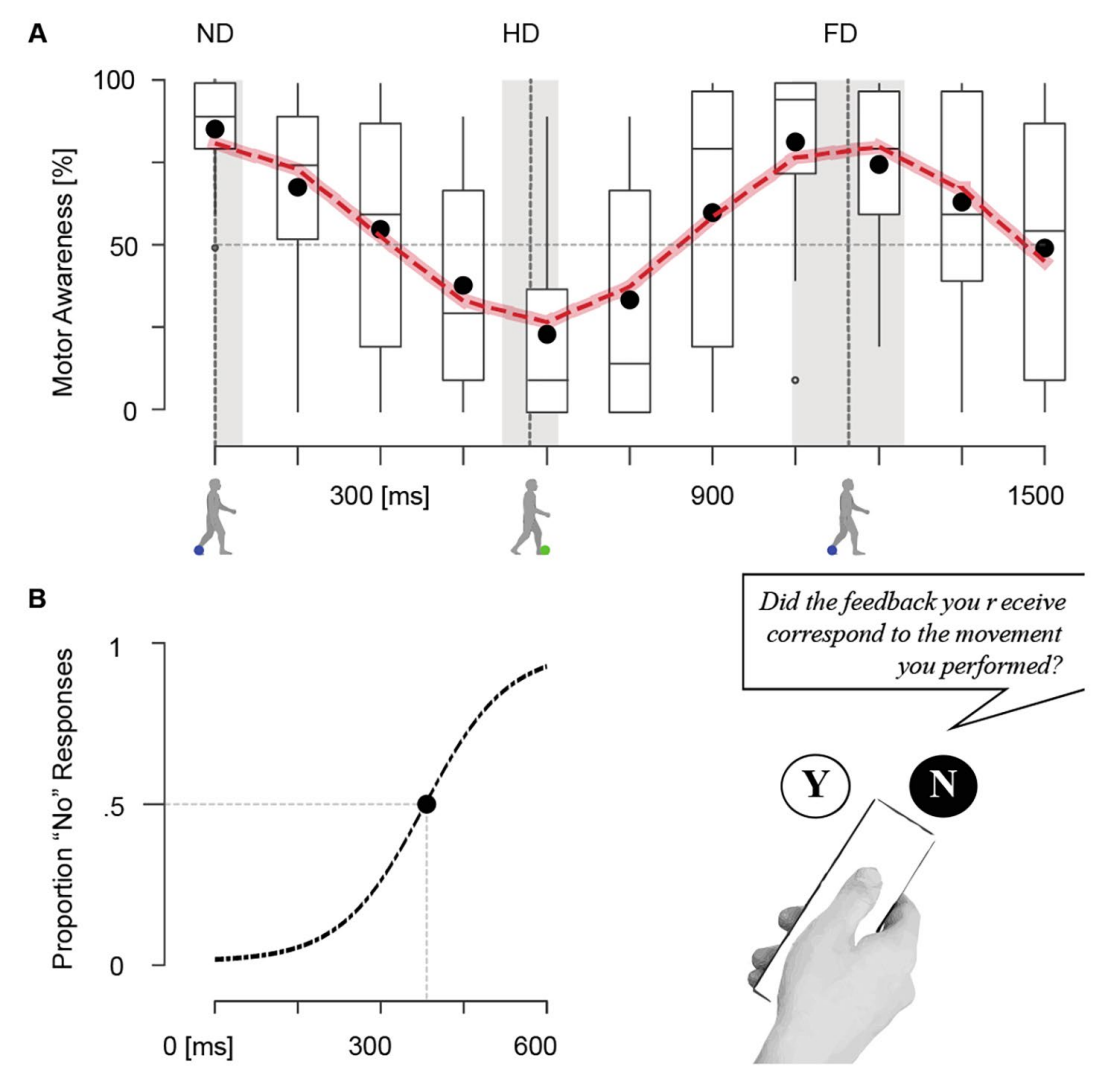
Motor Awareness judgments. (**a**) MA as a function of delay. ND corresponds to non-delayed trials, HD to a mismatch of a half-cycle step, and FD to a full step cycle. The vertical line indicates the average single step and full step-cycle, with their standard deviation underlaid in grey. The cosine function (dashed line) confirms the regularity of MA modulation (y-axis) as a function of the delay (x-axis). Black circles show the population’s mean MA ratings. Participants self-attributed the majority of trials for temporally synchronous feedback (ND, FD). MA judgments decreased with increasing delays until 600ms, which correspond to a half-step cycle delay. At HD trials, participants receive tactile feedback left-right reversed, resulting in the lowest self-attribution ratings. The boxplot of MA judgment across delays (median ± 1.5 interquartile range whiskers, 25th to 75th percentile range, and outliers). Boxplots indicate higher intersubjective variabilities for trials that are neither temporally nor spatially congruent. (**b**) Temporal thresholds were determined by fitting a cumulative Gaussian (cf. Data Analysis). The temporal threshold for remapped tactile walking agency was estimated as 378ms

## Results

### Motor awareness

Statistical analysis showed that MA ratings were significantly modulated as a function of delay (main effect of Delay: F(4.00, 100.04) = 16.08, *p* < 0.001, Greenhouse-Geisser correction applied). A cosine model showed a systematic modulation of MA as a function of the delay (Fig. 3a). The cosine model had a significantly lower residual sum of squares (i.e., the variability not explained by the model; F(2, 282) = 51, *p* < 0.001) compared to the linear model (38% reduction). Using BIC and AIC, we compared the goodness of fit for the linear and nonlinear models and found smaller AIC and BIC for the cosine model (AIC = 152, BIC = 170.9) compared to the linear model (AIC = 236, BIC = 247), showing that the cosine model is better at capturing the MA rating data. As illustrated in Fig. 3a, participants gave high MA ratings of 85 ± 3% for non-delayed trials (ND), in which step-related tactile feedback was provided close to real-time with respect to their actual stepping (60ms intrinsic delay). As hypothesized, self-attribution rapidly decreased with increasing delay.

We calculated a temporal threshold of 378 ± 239 ms (mean ± SE) (50% point of subjective equality; including the 60ms intrinsic delay; see Fig. 3b), so that for delays above this threshold, the majority of movements were judged to not correspond to the ongoing movement. The lowest self-attribution in MA was observed between 300-900ms with the minimum at 600ms (22 ± 6%), which is close to the half-cycle delay (µ = 571ms). In these trials, the actual movement and the remapped-tactile sensation are maximally out of phase so that the feedback is left-right reversed concerning the leading leg and heel-strike of the participant (i.e., left foot on ground and right foot providing feedback on the back). MA judgments increased again for larger delays peaking in trials with 1050ms delay (1110ms with 60ms intrinsic delay) that matched the participants’ step-cycles (1144 ± 9.1ms). Thus, MA for trials with a full step-cycle delay (FD) was high (78 ± 4%) and approximated the MA for ND trials. A Bayesian t-test provides evidence that MA for ND (0ms) and FD (1050ms) trials was comparable (MA_ND_≠MA_FD_, BF10 = 0.241).

**Table 1:** Gait parameters for the baseline walking conditions

	Stride-time	CV	Stance Phase		Swing Phase		Single Support		Initial DS		Terminal DS	
	[ms]	[%]	[ms]	[%]	[ms]	[%]	[ms]	[%]	[ms]	[%]	[ms]	[%]
**Left**	1136 ± 92	2.57 ± 0.33	645 ± 52	57 ± 3	486 ± 60	43 ± 3	481 ± 57	74 ± 8	86 ± 30	13 ± 4	83 ± 32	13 ± 5
**Range** **(L)**	969–1374	2.13–3.66	565–780		396–606		392–605		30–131		13–128	
**Right**	1136 ± 92	2.6 ± 0.28	654 ± 54	58 ± 3	482 ± 58	42 ± 3	486 ± 60	75 ± 9	83 ± 32	12 ± 5	86 ± 30	13 ± 4
**Range (R)**	969 –1374	2.11 –3.42	556 –791		392 –606		397 –608		10 –127		30 –132	

### Gait

**Fig. 4:**
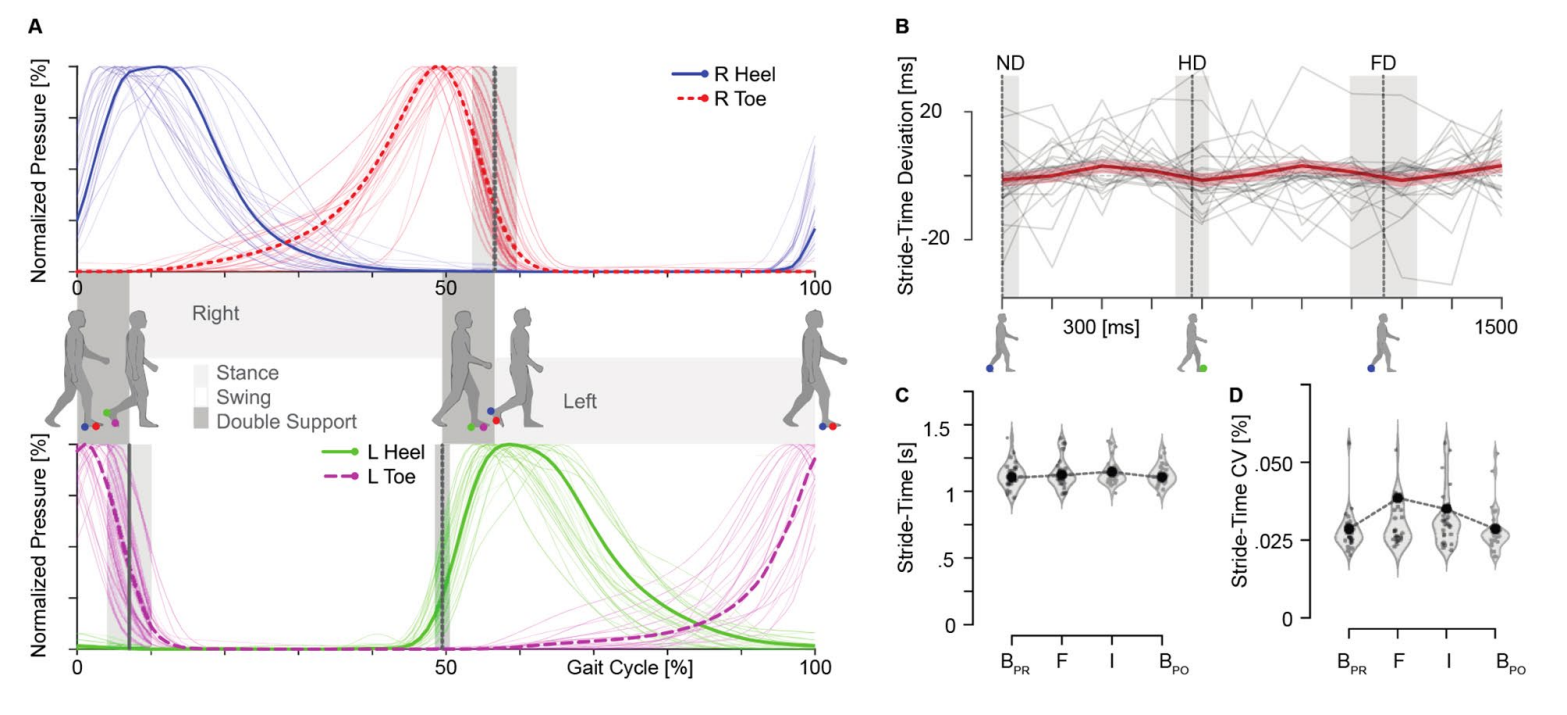
Gait Parameters. (**A**) Pressure Profiles. The insole data provide information about the pressure applied at the heel and front-foot and are used to normalize the gait-cycle. The solid blue line indicates the right heel sensor, which starts and ends the gait-cycle once a personalized threshold is crossed. The dotted red line is used to calculate the toe-off event of the right foot using the toe-sensor. (**B**) Gait adaptation. ND corresponds to non-delayed trials, HD to a mismatch of a half step-cycle, and FD to a full step-cycle. A sinusoidal function (red line) was fitted to the data (y-axis: stride time deviation, x-axis: delay), showing that the variation of stride time deviation is weakly systematic. Participants tend to walk slower for trials that are neither temporally nor spatially congruent. Grey lines in the background represent each individual’s data. (**C**) The violin plots indicate the distribution of the participants’ mean stride time (M_ST_) for three baselines (B_Pre_, B_Post_, and familiarization) and the intervention (Interv.) session. Participants’ M_ST_ did not significantly change across different blocks. (**D**) Stride time coefficient of variation (CV_ST_) for three baselines and the intervention block. While the CV_ST_ seems to be higher in intervention, no significant difference was observed across blocks

As listed in Table 1 all common temporal gait characteristics could be captured with the FeetBack system. Normalised pressure profiles are illustrated in Fig. 4a. For the main intervention session, only the stride-time data were analysed in the current cohort, Fig. 4 panels B-D. Participants’ gait period was, on average, 1146 ± 98ms (range: 986-1379ms), compatible with data in healthy participants [59] and data in a comparable task using auditory feedback [42]. While we did not observe a significant main effect of delay on stride-time deviation (F(5.37, 134.25) = 1.81, *p* = 0.11), a sinusoidal model of the stride time deviation has a significantly lower residual sum of square (F(2, 282) = 4.53, *p* = 0.01) compared to a linear model. This reduction is about 3% compared to the linear model. We further used the BIC and AIC to compare the goodness of fit for the linear and nonlinear models. We found slightly smaller AIC and BIC for the sinusoidal model (AIC = 2105.46, BIC = 2123.74) compared to the linear model (AIC = 2110.52, BIC = 2121.49), showing that the sinusoidal model was only minimally better at capturing the stride time deviation data. As illustrated in Fig. 4B, participants walked slightly faster than the average for trials with rhythmically synchronous feedback (i.e., ND, HD, and FD trials) and slower for those neither temporally nor spatially congruent (e.g., ¼ cycle, ¾ cycle, 5/4 cycle). Post-hoc analysis showed that stride time deviation remained stable in these ND, HD and FD trials (ND-HD: t = 0.59, *p* = 1; ND-FD: t = 0.34, *p* = 1; HD-FD: t = 0.24, *p* = 1).

In order to control for adaptation or carry-over effects of the vibration feedback on the walking characteristics we compared stride time between the pre and post baseline blocks (M_ST_, CV_ST_). Statistical analysis showed that M_ST_ and CV_ST_ did not significantly differ between feedback and no-feedback blocks (M_ST_: t(51) = 0.62, *p* = 0.54; CV_ST_: t(51) = 0.54, *p* = 0.58), Fig. 4, panels C and D. Finally, we did not observe any significant correlations between participants’ overall MA ratings and their average walking characteristics, including stride-time, stride-time deviation, or coefficient of variance (all *p* > 0.336).

## Discussion

In the current study we demonstrated that participants perceive tactile feedback, generated from each footfall but remapped to their own back, as corresponding to their own movement. This correspondence decreased when temporal delays were introduced up to the point when the feedback appeared left-right reversed as demonstrated in participants’ subjective reports of motor awareness. Further increasing the delay led to a re-synchronisation with the prior gait-cycle such that the feedback was again perceived to correspond to their own, on-going movement. We discuss our findings with respect to its implications for motor-neurorehabilitation.

### Motor awareness

While tactile feedback has been used in locomotor rehabilitation settings these prior studies have not systematically investigated to what extent participants perceive such feedback to be generated by, or correspond to their own, on-going movements. The focus was usually on investigating the effects of the feedback on spatiotemporal gait parameters. However, the perceived correspondence of such haptically augmented information may modulate to what extent individuals integrate feedback into their sensorimotor loop in a predictive manner [7–9]. In turn, this may contribute to gait adaptation, rehabilitation, and transfer-of-learning to activities of daily living. This latter point is particularly relevant with respect to understanding under what conditions movement adaptations observed under training conditions transfer to natural walking [67, 68] and how they transfer between effectors [69]. Corroborating previous findings in studies using visual and auditory gait-feedback [42, 44], we observed that participants perceived both synchronous as well as re-synchronised feedback as corresponding to their own movement. As reported for visual feedback, correct feedback lateralization mattered in the present conditions as temporally synchronous but left-right reversed trials were not perceived to correspond to the ongoing movement, but correctly as maximally out-of-phase.

Do the natural heel-strike and the remapped tactile feedback complement each other, or do they create an additional multisensory conflict? In the present study, we observed pronounced individual differences in MA ratings. Accordingly, MA thresholds were higher (378 ms) and noisier (i.e., higher variability; SD = 239 ms) for remapped tactile feedback compared to those reported in the auditory (∼ 200 ms) and visual (∼ 210 ms) gait paradigms (and auditory or visual agency studies in general; for review, see [70]). In prior studies, naturally occurring visual and acoustic feedback is blocked out either by using white noise or by obstructing the view of the lower limbs. In other words, feedback is substituted with experimentally controlled feedback in the same modality and relative location. In the current study, participants still perceived the actual ground reaction forces at each foot-strike, as it was not possible to remove the physical somatosensory action consequence. This results in an additional intra-modal (tactile) but spatially remapped conflict between touch on the foot sole and the back (missing in visual and auditory gait agency studies). Although participants were instructed to base their responses on the remapped tactile sensations on their back, their actual foot-strike may have still interfered with the present MA judgments. In terms of the central monitoring framework [23], which has been argued to depend on a comparison of internal representations and predictions about our movements with the actual reafferent sensory information (but also with our intended or desired state; see [19]), this corresponds to a strong conflict in the feedback source. Such uncertainty may additionally contribute to the higher temporal thresholds observed here along with potentially differing relative weighting of feedback cues [71].

Alternatively, two competing tactile sources may present a cognitive load affecting both walking characteristics and MA. The effects of a secondary perceptual, motor, or cognitive task (dual tasking) on the control of posture and locomotion are well-documented (for review see [72] and [73]), and usually manifested in an increase in stride time and increased gait variability [74]. Along these lines, [18, 39] reported that cognitive loading, via an articulated backwards counting task, suppressed gait synchronization and led to slower walking velocities during a gait ageny task. As discussed in these studies, the effect is most pronounced when the mismatch in the feedback is at its maximum. This suggests that resolving the temporal delay (or spatial deviation), as opposed to integrating the feedback appears to drive cognitive loading. In the current study, participants showed a tendency towards higher stride time variability and increased stride time when receiving remapped feedback (see Fig. 4c and d), although these differences did not reach statistical significance. In case of a clinical study, the effect of the remapped feedback should initially be evaluated by itself, as in the baseline condition here. This should provide an indication of the potential cognitive load and its effect on locomotor control. A comparison can then be made between walking without feedback, walking with synchronous feedback, and walking with systematically delayed feedback.

### Limited gait adaptation

Unlike our hypothesis and unlike previous work [42, 44], we did not observe a clear modulation of gait parameters. Participants stride-times and variability remained stable across all conditions and temporal delays. Why did participants not synchronise to the delayed feedback as described for some earlier studies? While gait-synchronisation has been previously observed, the effects are usually quite modest, particularly in healthy populations as in the current study. Our results are instead in line with studies on rhythmic stimulation, which reported no change in walking speed or cadence of healthy adults when they were asked to walk at their preferred pace [75]. Differences may further be due to the feedback modalities and stimulation parameters. Although MA thresholds are comparable across sensory modalities and even the effector (body part), our motor system is usually more sensitive to such differences [76]. For example, the sound generated by a heel-strike is temporally very discrete, whereas the tactile feedback continues throughout the entire stance-phase for each leg. Accordingly, the short sound bursts used in [42] differ from the vibrotactile apparent movement employed here. Such a rhythmical feedback may cause participants that are walking in incidental syncopation [52], that is, out-of-synch or off-beat with respect to their feedback, to adapt their gait and synchronise to the auditory cue [77, 78]. As discussed in [78], an initially off-beat, syncopated pattern in rhythmic sensorimotor behaviour “progressively loses stability until at a critical value […] of approximately 2Hz” where “the system undergoes a spontaneous transition to the synchronized pattern”. Such an automatic synchronization may be particularly relevant for human locomotion as the step frequency is usually around 2 Hz (1.75 Hz in the current study).

While both auditory and tactile approaches approximate an ecologically valid locomotor cue, it remains to be evaluated if a similarly short burst of tactile feedback may improve gait adaptation because of the increased temporal acuity, although it may be detrimental to the motor awareness and perceived correspondence of the feedback. This has for example been demonstrated for bidirectional (prosthetic) interfaces, where discrete tactile stimuli can dominate a multisensory percept, for better or worse [79], but that a biomimetic approach, mimicking natural feedback, and as proposed with our vibrotactile apparent movement here, improves aspects such as grasping performance and haptic perception [80]. The dampened synchronization reported here may also be related to the large inter-individual differences observed for MA. As discussed, this may be partially due to the fact that there are two tactile cues, the actual heel-strike and the remapped heel-strike. These cues can either correspond, as for (re-)synchronized feedback, or be in conflict as in the case of intermediate temporal delays and thus potentially interfere with adaptation. While the primary focus of the current study was to investigate MA, the setup could be used to target gait adaptation by increasing trial duration on a longer walking track. Furthermore, vibrating insoles could be used to blur the impact of the natural heel-strike, potentially shifting reliance towards the experimental, remapped feedback [71, 81]. Finally, a biomimetic approach may be compared to discrete tactile stimulation indicating heel-strike and toe-off events.

### Clinical application

Feedback in the form of external sensory cueing (i.e., auditory or visual) has extensively been investigated in PD and linked to improved gait characteristics (cadence, velocity, and stride-length) [82, 83]. This approach has been particularly promising with respect to tackling freezing-of-gait. Yet, challenges remain to demonstrate long-term consolidation of such advantages [84]. As touched upon in the introduction, some of these feedback systems have demonstrated that rhythmic tactile stimulation during locomotion may similarly improve gait characteristics in PD [12], stroke [13], and hemi- or paraplegic patients [14], as well as lower-leg amputees [15]. As our system can apply temporal delays that are individually adapted for each leg, it may also be used to investigate and modulate gait-asymmetry [85]. In the case of PD, gait impairments have been linked to an over-reliance on visual information [86], such that tactile feedback may provide a more salient cue immediately relevant to the on-going movement. Given our initial results, we aim to evaluate the FeetBack system in clinical populations and evaluate if and how their motor awareness may differ from healthy controls and if the the system can improve both their motor awareness and their gait by augmenting available [87, 88] or substituting lacking sensory information [89]. Furthermore, observing a relationship between the patients’ motor awareness, that is the perceived correspondence between actual and augmented tactile feedback, and their gait parameters may provide additional information on the causality between perception and action in sensorimotor adaptation.

## Conclusion

The present study investigated the contribution of haptic feedback on motor awareness and locomotor control in healthy participants. We demonstrated that remapped haptic feedback modulates MA in a systematic, predictable manner. Participants reported highest self-attribution for synchronized and re-synchronised trials and gave lowest ratings for trials with left-right reversed feedback, underlining the importance for lateralization. Although our findings are generally in line with previous gait agency studies, we observed a higher intersubjective variability in motor awareness and limited gait adaptation to the delayed remapped haptic feedback. Nonetheless, our results demonstrate a clear potential for using the FeetBack system to enhance gait awareness in patients with peripheral or central neuropathies as well as patients presenting sensorimotor symptoms in neurodegenerative diseases; populations that also stand to gain the most out of the feedback to normalize their walking characteristics.

## Data Availability

Study data have been deposited and are available on the Open Science Framework https://osf.io/5wy3v/.
